# Characterization of Human Type C Enterotoxin Produced by Clinical *S. epidermidis* Isolates

**DOI:** 10.3390/toxins10040139

**Published:** 2018-03-27

**Authors:** Chimène Nanoukon, Dissou Affolabi, Daniel Keller, Rodrigue Tollo, Philippe Riegel, Lamine Baba-Moussa, Gilles Prévost

**Affiliations:** 1 Institut de Bactériologie, 3 rue Koeberlé, Université de Strasbourg, CHRU Strasbourg, Fédération de Médecine Translationnelle de Strasbourg, VBP EA 7290, F-67000 Strasbourg, France; chimnanouk85@yahoo.fr (C.N.); dkeller@unistra.fr (D.K.); riegel@unistra.fr (P.R.); 2 Laboratoire de Microbiologie du Centre National Hospitalier et Universitaire Hubert Koutoukou-Maga, Cotonou 01 BP 386, Benin; affolabi.dissou@yahoo.fr (D.A.); tollorodrigue@yahoo.fr (R.T.); 3 Faculté des Sciences et Techniques, Laboratoire de Biologie et de Typage Moléculaire en Microbiologie, Université d’Abomey, Calavi, Cotonou 05 BP 1604, Benin; laminesaid@yahoo.fr

**Keywords:** enterotoxins, superantigens, coagulase-negative staphylococci, *S. epidermidis*, mitogenicity, cytokines

## Abstract

Staphylococcal Enterotoxins (SEs) are superantigens (SAg) originally produced by *S. aureus*, but their presence in coagulase negative staphylococci (CNS) has long been suspected. This study aims to better characterize a novel C-like enterotoxin expressed by clinical *S. epidermidis* strains, called SEC_epi_. We isolated and characterized SEC_epi_ for its molecular and functional properties. The toxin was structurally modeled according to its significant similarity with *S. aureus* SEC3. Most of SEC amino acid residues important for the formation of the trimolecular Major Histocompatibility Complex II MHCII–SEC–T Cell Receptor TCR complex are conserved in SEC_epi_. The functional properties of SEC_epi_ were estimated after cloning, expression in *E. coli*, and purification. The recombinant SEC_epi_ toxin exhibits biological characteristics of a SAg including stimulation of human T-cell mitogenicity, inducing and releasing high cytokines levels: IL-2, -4, -6, -8, -10, IFN-γ, TNF-α and GM-CSF at a dose as low as 3.7 pM. Compared to SEC_aureus_, the production of pro-sepsis cytokine IL-6 is significantly higher with SEC_epi_-activated lymphocytes. Furthermore, SEC_epi_ is stable to heat, pepsin or trypsin hydrolysis. The SEC_epi_ superantigen produced by CNS is functionally very close to that of *S. aureus*, possibly inducing a systemic inflammatory response at least comparable to that of SEC_aureus_, and may account for *S. epidermidis* pathogenicity.

## 1. Introduction

Staphylococcal superantigens (SAgs) represent a large family of at least 23 members in *S. aureus* that includes toxic shock syndrome toxin-1 (TSST-1), staphylococcal enterotoxins (SEs), and SE-like (SEl) where emetic activities are lacking, not yet confirmed or whose roles in diseases remain under investigation [1,2,3,4]. SEs are common causes of food poisoning but were also involved in acute atopic eczema [5], urticaria [6] and rheumatoid arthritis [7]. Their involvement in lethal sepsis, infectious endocarditis, acute kidney injury and necrotizing pneumonia are also demonstrated in animal models [8,9]. These toxins are not only active on gastrointestinal and endothelial cells, but also function as superantigens that stimulate a non-specific but vigorous proliferation of T-cells bearing certain T Cell Receptor Vβ-chain variable regions [2,10]. This hyperstimulation occurs through the binding of enterotoxin to the α-helical regions of the Major Histocompatibility Complex class II (MHC-II) molecules present on antigen-presenting cells outside the peptide binding sites of the normal antigens [10]. Thus, conventional antigens stimulate 0.01% of T-cells, while SAgs can stimulate 30% or more of T-cells, according to individual specificities [10]. SEs superantigenicity induces many shared activities among which are cytokine induction, pyrogenicity, lymphocyte proliferation, immunosuppression, and toxic shock. Due to these properties, SEs are assumed to be a threat for public health.

In coagulase negative staphylococci (CNS), there are only few studies that demonstrate their ability to produce stable enterotoxins. Recent studies indicate that CNS isolated from veterinary sources and food may produce typically *S. aureus-*related SEs [11,12], but not CNS from human sources [13]. However, it has been recognized that superantigen genes are often associated with mobile genetic elements (MGEs), such as pathogenicity islands, prophages, or plasmids [14,15]. This suggests that SEs genes can move from *S. aureus* strain to strain by horizontal transfer and that the colonization of the skin and the mucosa, simultaneously by several *Staphylococcus* species might promote such genes transfer from *S. aureus* to skin commensal CNS. The *sec* gene encoding enterotoxin C has been reported among the most common enterotoxins observed in clinical *S. aureus* after, SEA, SED et SEB [16]. This toxin is produced by 16% of clinical *S. aureus* isolates [17]. Three major subtypes of SEC toxin (SEC1-3) exist. SEC3 differs from enterotoxins SEC1 and SEC2 by four and nine amino acids, respectively [16]. The three subtypes are immunologically close [18,19] and all activate T lymphocytes though T Cell Receptors (TCR) bearing hypervariable regions Vβs 3, 12, 13.2, 14, 15, 17 and/or 20 [20]. Nevertheless, it was shown that SEC2 and SEC3 have more affinity for TCR that bears Vβ 13.2 but bind more weakly TCR than SEC1 to TCR bearing Vβ3 [20]. This difference is due to a valine substitution at position 26 in SEC1 by a tyrosine in SEC2 and SEC3 [20]. In the CNS group, *Staphylococcus epidermidis* is one of the species that has attracted most attention by their involvement in serious hospital infections, particularly in cases related to prosthetic joint surgery [21,22]. A single human clinical *S. epidermidis* strain was previously characterized for carrying *sec* gene [23]. We previously reported the production of a staphylococcal enterotoxin C analogous in two new clinical *S. epidermidis* SE90 and SE95 isolates from Africa [24]. In the present study, we purified the novel type C-like enterotoxin produced by *S. epidermidis* named SEC_epi_ to determine its molecular, biological, and immunological characters as a preamble of its potential involvement in the pathogenicity of *S. epidermidis*.

## 2. Results

### 2.1. Characteristics of the Putative sec_epi_ Gene Encoded by Clinical S. epidermidis Strains

**Figure 1:**
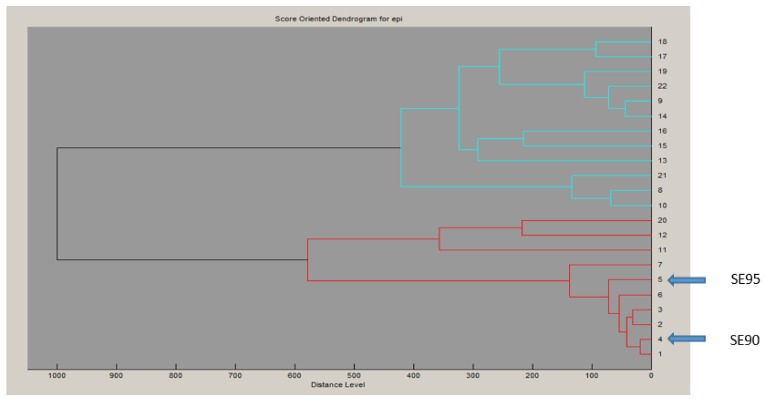
Proteomic relatedness. Dendrogram generated by Biotyper™ based on MALDI-TOF spectral profiles of *S. epidermidis* isolates.

**Figure 2:**
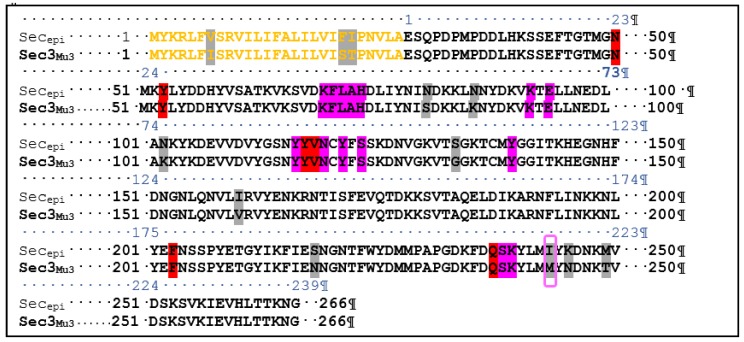
ClustalW alignment of amino acids sequences between *S. epidermidis* SEC3 (SEC3_epi_) and *S. aureus* Mu3 SEC3 (SEC3_Mu3_). Putative signal peptides are colored in yellow. The non-corresponding amino acids, amino acids required for binding to TCR Vβ chain and residues contacting MHC II (HLA-DR) are highlighted in grey, red and pink, respectively. The non-corresponding residue SECepi I216 to binding site of MHC is surrounded by a pink frame. Genomes of the *S. epidermidis* strains 90 and 95 are registered with the codes CP024408 and CP024437, respectively [25].

To examine the superantigenic capacity of the clinical *S. epidermidis* strains, we previously screened 22 *S. epidermidis* isolates from various clinical samples for enterotoxins genes (*sea*, *seb*, *sec*, *seg* and *seh*) by polymerase chain reaction (PCR) using specific primers. Two isolates (9%) from blood were found to carry and to express *sec* [24]. When using radial immunoprecipitation method and a standard range of purified toxin concentrations (data not shown), *sec*_epi_ is expressed at 60–90 µg/mL by both SE90 and SE95 isolates culture supernatants after 24 h of growth at 37 °C. The *sec* gene was sequenced from these two *S. epidermidis* isolates. Homology analysis of the deduced amino acids sequences of the amplified *sec* genes revealed that SEC_epi_ were 100% conserved among both two isolates, while analysis of the generated dendrogram issued from the MALDI-TOF spectrum of the 22 strains shows that both the two *sec* positive isolates strains do not aggregate in a single cluster (Figure 1). We amplified through PCR the corresponding Open Reading Frame (ORF) and sequenced the designated *sec*_epi_ encoding *S. epidermidis* staphylococcal enterotoxin-like toxin C (SEC_epi_). The corresponding peptidic chain is 266 amino acids long for a structural gene of 801 bp (Figure 2). Thus, SEC_epi_ is closely-related to previously described SEs, albeit very efficiently secreted. Based on the results of homology comparison between the peptide chain of SEC_epi_ and known *sec* gene sequences, we found that *sec*_epi_ is close to *S. aureus* Mu3 *sec*3 gene, with three amino acids substitutions in the signal peptide (I7V, S21F, T22I), albeit efficiently secreted, and nine amino acids substitutions in the mature protein (S54N, K59N, K75N, G106S, V133I, N191S, M216I, N218K, T222M) (Figure 2). Furthermore, several SEC amino acid residues are important to the formation of the trimolecular MHC–SEC–TCR complex and, thus, promoting superantigenicity (amino acids for binding to TCR colored in red and amino acids for binding to MHC colored to pink), and conserved in the SEC_epi_ protein (Figure 2). We noted that only methionine 216, participating in the binding of MHC (surrounded by a pink frame) was substituted by an isoleucine in SEC_epi_. Using the online signal peptide prediction software Expasy Compute pI/Mw tool, we predicted the N-terminal amino acids sequence of the mature form of SEC_epi_. The putative secreted protein sequence of SEC_epi_ has 239 amino acid residues with a predicted molecular weight of 27.6 kDa, and an isoelectric point at 6.35, while a disulfide bridge is formed, and, thus biochemical properties similar to those from *S. aureus* SEC.

### 2.2. Structural Features of SEC_epi_ and Structural Homology with SEC_aureus_

**Figure 3:**
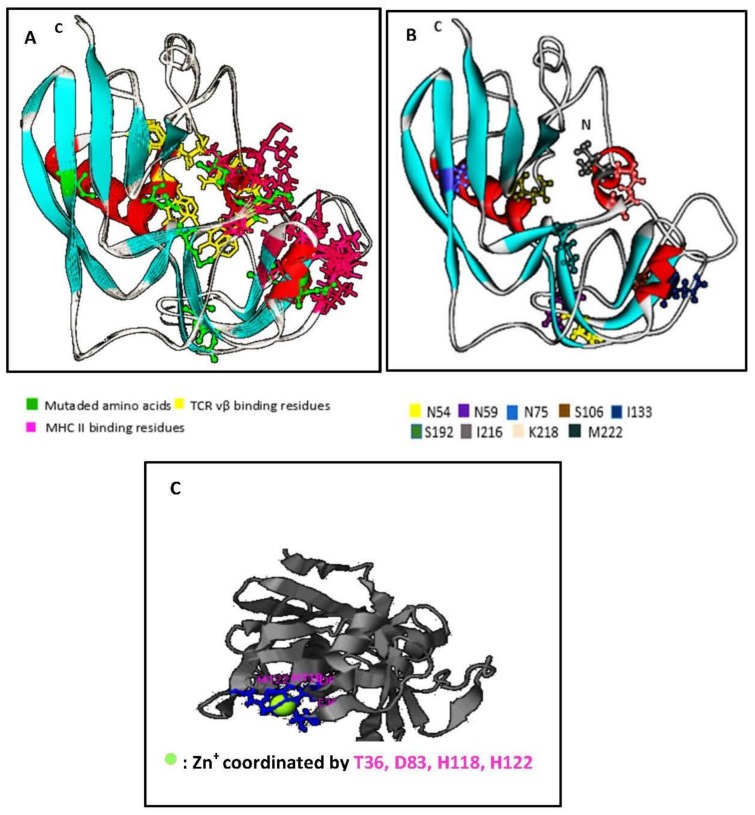
Prediction of the structure model of SEC_epi_, using I-TasserTM and formatted through Weblab viewer Pro 3.7, and structural alignment and zinc binding sites; (**A**) Structure model of SEC_epi_. The predicted conformation was determined as “superantigen” in reference to its remarkable sequence. The resulting conformation is close to that of other superantigens 3D structures. The mutated amino acids are in ball and stick; (**B**) Structural alignment of SEC_epi_. Superimposition of the structure of SEC*_aureus_* (in line ribbon) with the predicted structure of SEC_epi_ (in solid ribbon) shows that both toxins significantly matched together. The TCR binding sites (in yellow stick) and MHC binding sites (in pink stick) of both SEC isoforms are well superimposed. The mutated amino acid residues in SEC_epi_ are colored in green ball and stick; (**C**) ligand binding sites of SEC_epi_, the zinc atom is represented as a green ball. Ligand binding sites residues identified in this study are colored in green stick.

Using I-Tasser™ and WebLab ViewerPro™ software, we generated a predicted SEC_epi_ 3D structure. The predicted conformation of SEC_epi_ is characterized by unequal sized domains, as found too, in other bacterial superantigens. The larger domain top left is the β-grasp fold consisting of four to a five strand β-sheet over, which is in front of an α-helix. The small domain bottom right is the fold of a Greek-key β-barrel capped by an α-helix at its end and contains the disulfide loop (Figure 3A). A structural alignment of the predicted form of SEC_epi_ with the previously determined structure of *S. aureus* SEC type 3 in both I-TASSER™ software and in TM-align structural alignment program revealed that the amino acid residues involved in TCR and MHC bindings are structurally identical despite a M216I polymorphism (Figure 3B). A zinc coordination site is found in the structure of SEC_epi_ and is coordinated by T36, D83, H118, H122 residues (Figure 3C). The zinc-binding site residues observed in the SEC3_aureus_ crystal structure (deposited in the Protein Data Bank with the accession code 1ck1) vary with the amino acid D9 replaced by T36. This clearly shows a high structural homology between the two isoforms.

### 2.3. Expression and Purification of rSEC_epi_

**Figure 4:**
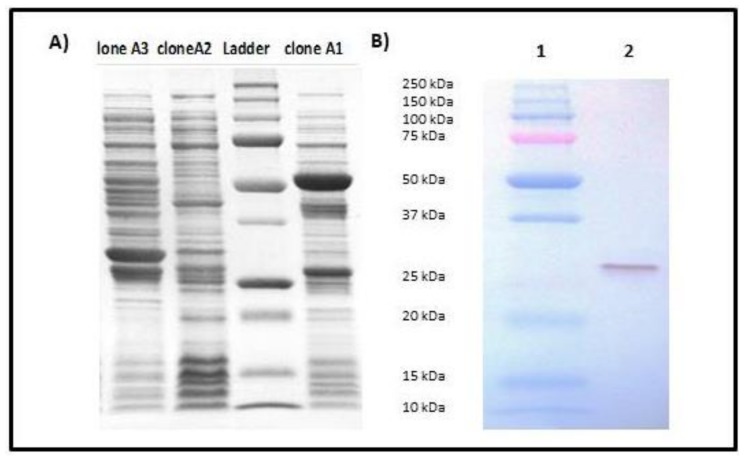
Analysis of cloning and identity of SEC_epi_ by 12% SDS-PAGE with Roti^®^-Blue staining. (**A**) SDS-PAGE 12% of the bacterial lysates of clones A1, A2 and A3 after cloning of *sec*_epi_. The red arrow indicates the 53 kDa band corresponding to SEC_epi_-GST expression by clone A1; (**B**) Immunoblotting of purified SEC_epi_ using rabbit affinity-purified *S. aureus* anti-enterotoxin C antibodies. Lane 1: Molecular weight standard, lane 2: 50 ng of rSEC_epi_.

Transformation of the recombinant vector pGEX-*sec*_epi_ into *E. coli* BL21 allowed us to obtain three A1, A2 and A3 clones resistant to ampicillin. The expression of the fusion protein SEC_epi_-GST in these clones was screened by SDS-PAGE after induction with the IPTG. The results showed that only clone A1 expresses SEC_epi_ by the presence of an approximately 53 KDa fusion protein of the GST (26 kDa) coupled to SEC_epi_ (27 kDa) (Figure 4A). After confirmation by nucleotide sequencing and peptide alignment with the SE90 and SE95 *sec*_epi_ gene amplicon sequences, recombinant SEC_epi_ was overexpressed in *E. coli* BL21 and purified by glutathione-Sepharose 4B chromatography. The immunological reactivities of rSEC_epi_ with rabbit affinity-purified *S. aureus* anti-enterotoxin C antibodies were assayed by using Western blot. As shown in Figure 4B, the antibody reacted with purified rSEC_epi_. These results indicate that antigenic relationships of rSEC_epi_ are close to SEC_aureus_.

### 2.4. SEC_epi_ Is Stable to Heat Treatment and Digestive Enzymes Treatment

**Figure 5:**
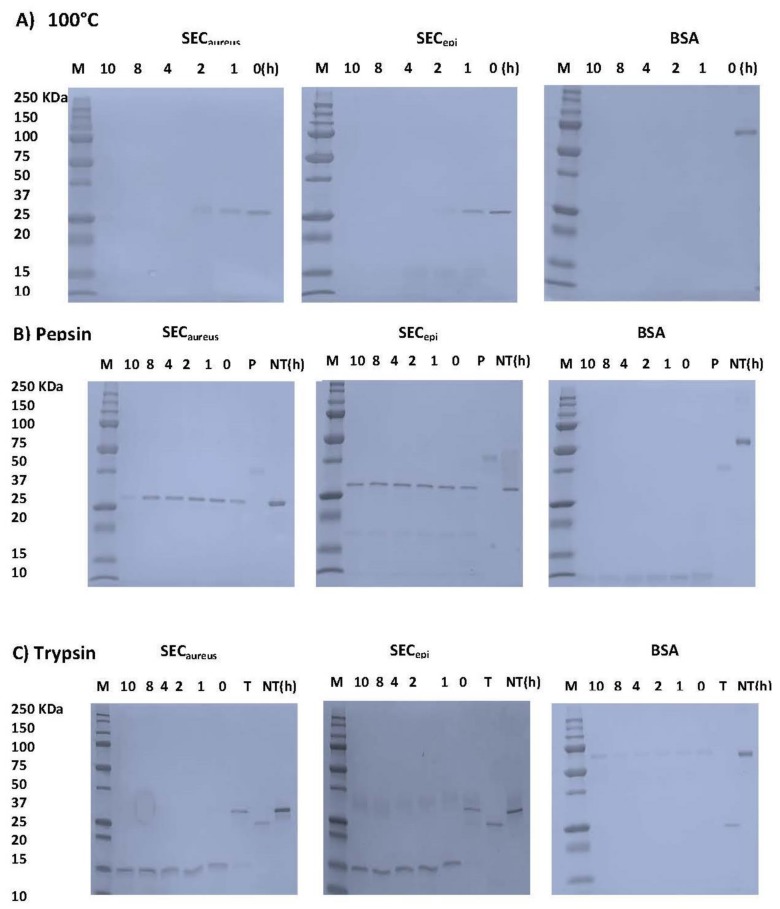
Effects of heat and digestive enzymes treatment on SEC_epi_ and SEC_aureus_. (**A**) enterotoxins (3.7 µM) were maintained at 100 °C. After the heat treatment, each sample was analyzed by SDS-PAGE; (**B**) Each protein at 3.7 µM was incubated with 0.028 µM of pepsin and analyzed by SDS-PAGE; (**C**) Each protein, at 100 µg/mL, was incubated with 0.04 µM of trypsin and analyzed by SDS-PAGE. BSA was used as negative control; NT, no treatment; P, pepsin; Tr, trypsin.

In this study, we examined whether SEC_epi_ resists to heat related to food preparation, pepsin, and trypsin digestion in gastrointestinal conditions. After heat treatment at 100 °C, 1–12 h, the samples were analyzed by SDS-PAGE (Figure 5A). The BSA band immediately disappeared before 1 h treatment. However, the SEC_epi_ and SEC_aureus_ bands persist after 1 h and before to become almost invisible at 2 h of treatment. For proteolytic enzymes analysis, the BSA band was no more visible 1 h after pepsin treatment. In contrast, the SEC_epi_ and SEC_aureus_ bands were still observed at up to 10 h after pepsin treatment and each showed a single band of equal intensity corresponding to its authentic size (27 kDa) (Figure 5B). Trypsin is the most important digestive protease in intestine of humans and animals. The stability of both enterotoxins upon trypsin digestion was further evaluated. SEC_epi_ and SEC_aureus_ treated with trypsin appeared as bands of molecular weight reduced by half (13 kDa) compared to the normal weight of the toxin in the SDS-polyacrylamide gels. However, these bands were observed until 12 h after treatment (Figure 5C). In contrast, trypsin-treated BSA appears as almost invisible bands already after 1 h (Figure 5C). Regarding the rate of hydrolysis, the trypsin hydrolyzed products generated by SEC_epi_ hydrolysis were identical to those generated from SEC_aureus_. This indicates that the mutations observed in the amino acid sequence of SEC_epi_ do not affect its resistance to degradation by heat, or some of the harsh conditions of the gastrointestinal tract, at least.

### 2.5. Functional Analyses of SEC_epi_

**Figure 6:**
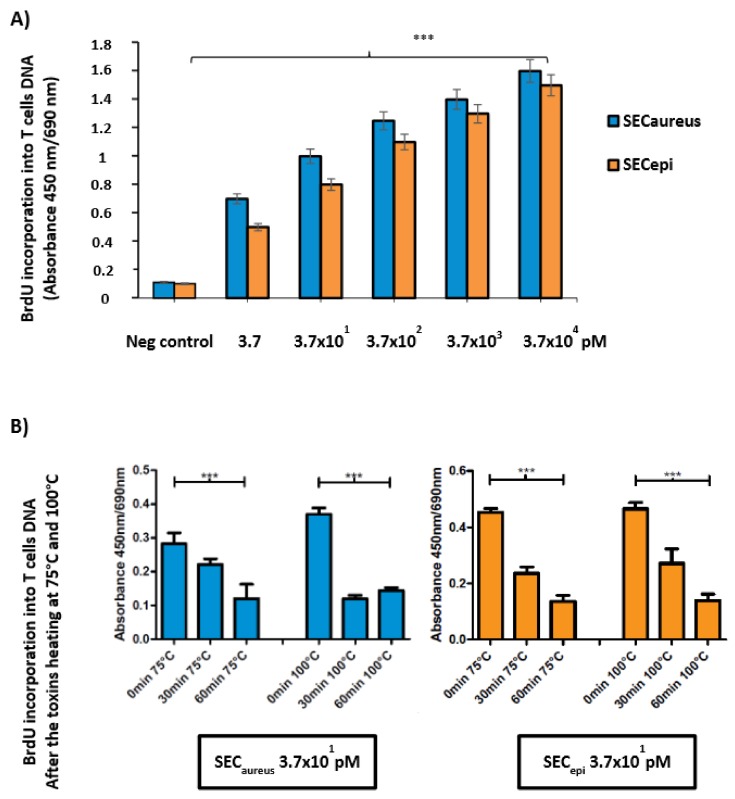
SEC_epi_ stimulates proliferation of human lymphocytes. (**A**) The proliferation of human PBMC activated with recombinant SEC_epi_ (orange bars) or SEC_aureus_ (blue bars) was measured by incorporation of 5-bromo-2′ deoxyuridine. Each bar represents the mean ± standard error of three independent experiments. Neg control: Negative control containing only PBMC; *** The results were significantly different in comparison with the negative control (*p* ≤ 0.001); (**B**) The proliferation of human PBMC activated with recombinant SEC_epi_ or SEC_aureus_ was measured by the incorporation of 5-bromo-2′ deoxyuridine after heat exposure at 75 °C and 100 °C according to time intervals of 0 to 60 min. The mitogenic activity of both toxins was significantly persisting after heating at 75 °C and 100 °C.

To examine the function of SEC_epi_ in *S. epidermidis* pathogenicity from human, a recombinant protein was purified from *E. coli* and used to assess the superantigenic activity of enterotoxin. Lymphocyte proliferation and cytokines production were measured using human PBMC from three healthy donors. First, PBMC were stimulated in the presence or absence of various concentrations of rSEC_epi_ and SEC_aureus_. SEC_epi_ was tested for mitogenic activity on stimulated human PBMC in a 5-bromo-2′ deoxyuridine (BrdU) colorimetric incorporation assay [15]. Figure 6 shows representative results of three experiments. Addition of 100 U of polymyxin B as a LPS inhibitor did not influence the results, indicating that the effect due to LPS contamination was negligible (data not shown). The toxin strongly induces intense human T-cells proliferation at concentrations close to 3.7 × 10^4^ pM (Figure 6), but the protein was significantly mitogenic at doses as low as 3.7 pM. Interestingly, the superantigenic activity of SEC_epi_ in human T cells is comparable to that of *S. aureus* SEC (Figure 6A). Moreover, exposure of SEC_epi_ and SEC_aureus_ at 75 °C and 100 °C to other time intervals of 0–60 min after cells T stimulation shows a decrease of mitogenic effect according to time, albeit the toxin conserved a mitogenic potential after 1 h treatment at 100 °C (Figure 6B).

**Figure 7:**
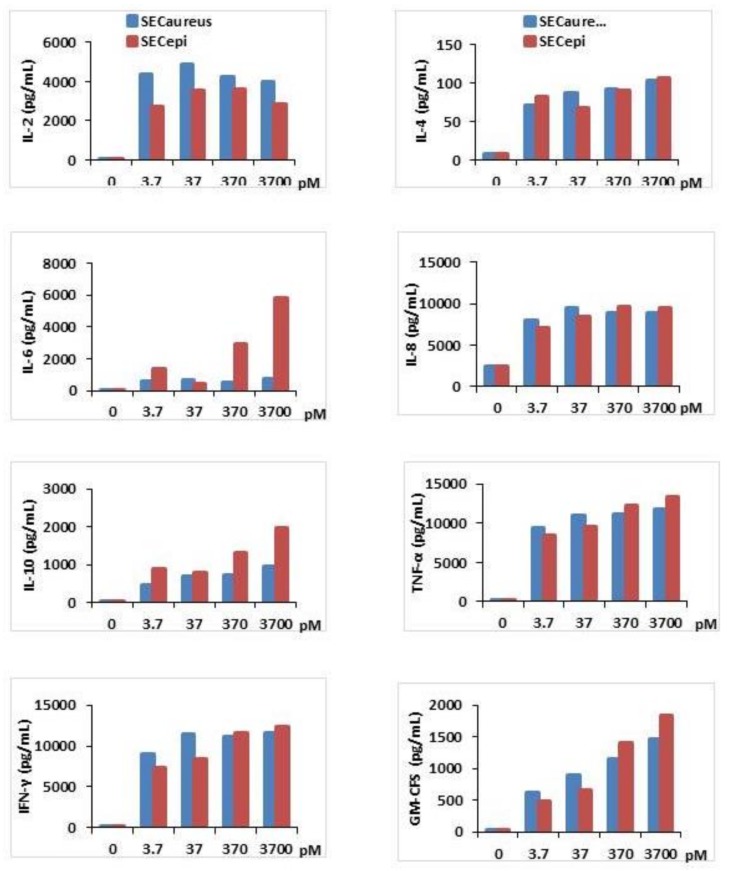
Cytokines production in T cells activated with recombinant SEC_epi_ (orange line) or SEC_aureus_ (Blue line) in vitro. T cells (2 × 10^6^ cells/mL) were prepared from three different donors and incubated for 72 h with concentrations ranging from 3.7 pM to 3.7 × 10^3^ pM of SEC_epi_ or SEC_aureus_. IL-2, IL-4, IL-6, IL-8, IL-10, IFN-γ, TNF-α and GM-CSF production in the supernatants of the T cells culture were determined by an ELISA sandwich. The results are representative of three experiments.

Second, the proinflammatory (IL-2, IL-6, IL-8, TNF-α, IFFN-γ, GM-CFS) and suppressive cytokines (IL-4, IL-10) titer in human PBMC stimulated by SEC_epi_ was measured and compared with that stimulated by SEC_aureus_ (Figure 7). The results showed that compared to the negative control, SEC_epi_ induced a strong production of all cytokines tested with dose of toxin as low as 3.7 pM. The cytokines concentrations obtained in the supernatants varied according to the nature of cytokines tested. As examples, TNF-α, IFN-γ, IL-8, Il-6, IL-2 are produced at high titers of up to 8000 pg/mL, 7000 pg/mL, 7000 pg/mL, 3000 pg/mL and 2500 pg/mL, respectively, after activation of T cells with 3.7 pM of SEC_epi_, while the same concentration of toxin stimulates the production of IL-10, GM-CSF, IL-4 to respective titers of 800 pg/mL, 500 pg/mL and 80 pg/mL (Figure 7). The comparison of cytokines production profile in human PBMC stimulated by SEC_epi_ with that of the wild-type (SEC_aureus_) shows that there is no significant difference between released quantities of most cytokines induced by the two toxins, *p* > 0.05. However, the induction of pro-sepsis cytokine IL-6 is significantly higher in lymphocytes, when activated by SEC_epi_ (*p* ≤ 0.01). Moreover, the lymphocytes stimulated by 3.7 × 10^3^ pM of SEC_epi_ are able to secrete up to 9–10 fold more IL-6 than those stimulated with the same amount of SEC_aureus_ (7500 pg/mL vs. 800 pg/mL, *p* ≤ 0.0001), suggesting that systemic inflammatory response induced by SEC_epi_ would be at least as severe than that caused by SEC_aureus_.

## 3. Discussion

Although it was reported that acquisition of SE(s) gene(s) by CNS strains would be a rare or isolated event [26], the present study revealed that the SEs genes exist not only in a food poisoning isolate [1], but also in CNS isolates from cases of human infectious diseases. Indeed, this SECepi is produced at significant quantities in culture supernatant of some *S. epidermidis* isolates. This work reports a strong sequence similarity between C-types staphylococcal enterotoxin sequence of clinical strains of *S. epidermidis* SE90 and SE95 named SEC_epi_ from Africa and wide-type *S. aureus* SEC3. This level of sequence similarity served as a benchmark for the determination of the mature sequence of SEC_epi_ as well as its structure and the amino acid residues involved in the biological activity of this toxin. ClustalW analyses between SEC_epi_ and its isoform *S. aureus* SEC3 reveals an evolutional signature corresponding to substitutions of several amino acids in the signal peptide and the secreted sequence of SEC_epi_ (I7V, S21F, T22I and S54N, K59N, K75N, G106S, V133I, N191S, M216I, N218K, T222M) (Figure 2). The prediction of the modeled SEC_epi_ structure produced an architecture close to the SEs [2,27] that has high structural homology with the predetermined structure of *S. aureus* SEC3 [28]. Moreover, the SE90 and SE95 SEC gene sequences is 100% identical to that of the FRI909 strain isolated in the United States [24], albeit at least the two first isolates are not strictly identical. This suggests that similar mechanisms of molecular evolution and acquisition of the *sec* gene originally transcribed by *S. aureus* might occur in the genome of *S. epidermidis* isolates from different geographical areas, Africa and America.

Because SEC_epi_ exhibited a high degree of identity of amino acid sequence and ligand binding sites (Figure 2 and Figure 3) with SEC_aureus_, we hypothesized that both toxins have very close biological properties. Using a signal peptide predictor software, we identified the mature SEC_epi_ sequence which was then cloned into pGEX-6P-1. The resulting recombinant protein (rSEC_epi_) was expressed and purified by affinity chromatography and the identity of the protein is confirmed by Western blot method as a ~27 kDa protein using rabbit affinity-purified *S. aureus* anti-enterotoxin C antibodies (Figure 4B) [28]. SEs are well-known to resist heating and digestive enzymes [28,29,30]. rSEC_epi_ was able to keep 30% function after heating at 100 °C for 1 h as SEC_aureus_ does (Figure 5 and Figure 6), more or less, but it was less resistant than SEA, which resists beyond 8 h of heat treatment [15]. In the case of gastrointestinal enzymes, the toxin was stable to pepsin and slightly unstable to trypsin action. There was no major alteration in SEC_epi_ susceptibility to pepsin and trypsin hydrolysis compared with the native SEC_aureus_. A comparison between the proteolytic enzyme stability of SEC_epi_ and SEA, the most studied enterotoxin, showed that SEC_epi_ is less resistant to trypsin activity than SEA, but has the same pepsin resistance profile as SEA [30]. In our previous study, 9% (2/22) of *S. epidermidis* clinical isolates originating from Benin secreted a functional SEC parent [24]. This frequency is even greater than in other reports, where only 3% (1/32) of *S. epidermidis* strains from food produced a stable enterotoxin C, while none of 200 clinical strains produced it [11,27]. In one of our studies involving 30 *S. epidermidis* strains from a significant clinical origin collected in the Microbiology laboratory of the Strasbourg hospital, there was no positive strain for the *sec* gene. However, approximately 16% of clinical *S. aureus* strains are producing SEC_aureus_ [17].

rSEC_epi_, which shows homology to SEC_aureus_, acts as a superantigen for human T lymphocytes because of its ability to induce lymphocytes proliferation and cytokines production from human T cells already with a low dose of the toxin (3.7 pM) (Figure 6 and Figure 7). Substantial amounts of proinflammatory cytokines such as TNF-α, IFN-γ, IL-8, IL-6, IL-10 and IL-2 were found in the isolated human T lymphocytes culture supernatants activated with SEC_epi_, while they were almost not detected in the culture supernatant of unactivated cells. Moreover, IL-6, which is an important mediator of the systemic inflammatory response in septic shock, is produced at a higher level in the culture supernatants of SEC_epi_ stimulated T cells [31,32]. In fact, high levels of IL-6 were found to predict a fatal outcome in patients with septic shock [33]. This increased cytokine concentration may accentuate the intensity of the immune response and result in a systemic shock leading to disseminated intravascular coagulation that can lead to hemorrhage, severe coagulation disorders and to the failure of organs, eventually leading to death [34]. The ability of rSEC_epi_ to stimulate human lymphocytes implies that this enterotoxin can bind to TCR Vβ elements and/or MHC class II molecules in the human [2]. In previous studies, several amino acid residues important for binding to TCR Vβ and MHC class II elements to SEC have been identified [35,36,37]. Except polymorphism at M216I, not any mutation of the involved amino acids is present in the peptide sequence of SEC_epi_ (Figure 3B), and this would support the preservation of the superantigenic activity of the secreted enterotoxin as well as the TCR Vβ epitope segregation. It remains hypothetical whether the substitution of Methionine at position 216 by Isoleucine in SEC_epi_ TCR Vβ binding site, is involved in a variation in the amount of IL-6 produced by the activated lymphocytes. We also found that through the binding area, which forms the zinc binding site of SEC_aureus_, only D9 of *S. aureus* is replaced by T36 in SEC_epi_ zinc binding amino acid residues sites (Figure 3C). A previous report noted that zinc binding is not essential for T cell stimulation, emesis, or lethality in SEC sub-types [27]. These findings are consistent with the fact that a mutation occurring at the usual zinc binding sites in SEC_epi_ does not alter its functions, as observed for *S. aureus* enterotoxin C2 [38].

Moreover, in this study, *S. epidermidis* SE90 and SE95 produced up to 100 μg/mL of staphylococcal enterotoxin C in 24 h at 37 °C of microbial broth culture. This dose largely exceeds the quantities of SEC_aureus_ found in 24 h culture supernatants (BHI) of *S. aureus* isolates FRI137 (296 ng/mL), FRI913 (4134 ng/mL) and a food strain, *S. epidermidis* 4 s (14 ng/mL) in another study [11]. Therefore, it might be possible that a similar amount of Sec_epi_ may be secreted in food contaminated with enterotoxigenic *S. epidermidis* since, in most of the food poisoning cases caused by SEs produced by *S. aureus*, where amounts ranging from 5 to 100 ng/g enterotoxins can be detected in the associated foods [39]. This suggests that SEC_epi_ would also be able to trigger a food poisoning outbreak. In addition, heating at 75 °C and 100 °C did not inhibit the activity of the toxin but could reduce their mitogenic effect on T cells.

These results, together with our previous data, demonstrate that clinical strains of CNS can also carry dreaded virulence factors in *S. aureus*, including the staphylococcal enterotoxin C. This study not only suggests a structural similarity between SEC_epi_ and SEC_aureus_ but also provides proof that SEC_epi_ may act as a superantigen in the human host although SEC wild-type carries several differences in *S. epidermidis*. Given the great similarity between SEC_aureus_ and SEC_epi_, the ability of SCN to produce enterotoxins in significant quantities also poses a real problem for food safety and may be some clinical cases. It now remains interesting to study in vivo toxicity and emetic activity of SEC_epi_, because the International Nomenclature Committee for Staphylococcal Superantigens [40] proposed that only staphylococcal superantigens inducing emesis after oral administration in an experimental model of primates should be designated as staphylococcal enterotoxins.

## 4. Materials and Methods

### 4.1. Ethical Statement

Buffy coats from fresh human blood from healthy donors were purchased from the « Etablissement Français du Sang de Strasbourg, France », for which all individual information remains confidential.

### 4.2. Bacterial Strains, Vectors Media, and Growth Conditions

*S. epidermidis* strains were clinical isolates originated from blood samples and identified with MALDI-TOF Biotyper™ (Bruker Daltonics) and 16S rDNA genes sequencing [24]. Dendograms were generated from main spectra projection using MALDI/TOF MS Microflex™ system and Biotyper™ software. Each *S.* *epidermidis* isolate was previously screened for SEA, SEB, SEC, SEG, and SEH by Western blot and immunodiffusion techniques [24]. *S. epidermidis* isolates that were called SE90 and SE95 were found to produce SEC and were chosen for further analysis of the enterotoxin. *S. aureus* SCP FRI 361 strain, a SEC producer was used as a positive control to verify the purified recombinant protein identity.

*Escherichia coli* XL1 blue cells [*recA1 endA1 gyrA96 thi1 hsdR17 supE44 relA1 lac* (F′ *proAB lacIqZΔM15 Tn10* (*tet*r))] (Stratagene, Agilent technologies, Massie, France) were used as recipient cells for transformation with recombinant pGEX-6P-1- *sec*_epi_ gene (GE healthcare life Science, Buc, France). *Escherichia coli* BL21 [F-, *ompT*, *hsdS* (*rB*-, *mB*-), *gal*] was used for over-expression of the glutathione-S-transferase (GST)-*S. epidermidis* enterotoxin C fusion gene, according to the manufacturer’s (GE healthcare).

For routine culture, bacteria were grown on Columbia blood agar plates (Oxoid). Overnight cultures were prepared in tryptone broth containing 4% yeast extract (Oxoid). 2% yeast extract-trypton broth (YT) was used for culturing *E. coli* BL21, *E. coli* XL1 and *E. coli* transformants. All cultures were incubated in an aerobic atmosphere at 37 °C.

### 4.3. DNA Isolation

Total DNA of *S. epidermidis* was purified using MasterPure™ DNA Purification according to the manufacturer’s recommendations (Epicentre, Le Perray en Yvelines, France). DNA purity was checked by a 260/280 ratio and samples with ratios < 1.8 were rejected. EZ-10 spin miniprep kit (Euromedex, Souffelweyersheim, France) was used for amplicons purification following the manufacturer’s instructions. For plasmid DNA isolation, *E. coli XL-1* plasmid DNA midi-preparation was performed with GeneJET™ Midiprep Kit (Thermo Scientific, Illkirch-Graffenstaden, France).

### 4.4. Nucleotide Sequencing and Analysis

Nucleotide sequencing of *sec* gene from *S. epidermidis* SE90 and SE95 (*sec*_epi_) and recombinant *sec*_epi_ gene was determined by sequencing through Sanger methods employing the dideoxynucleotide sequencing methodology [41]. Briefly, the *sec*_epi_ gene was amplified by PCR for the two strains of *S. epidermidis sec*^+^. Cinq μL (60 ng/μL) of purified amplicon were supplemented by 5 μL of oligonucleotide (5 μM) and sent to GATC (GATC BIOTEC, Konstanz, Germany) for sequencing.

The plasmid containing the intact sequence of rsec_epi_ was purified after *E. coli xl1* plasmid DNA midi-preparation. Ten microliters (80–100 ng/mL) of purified plasmid were added to 5 µL (5 pM) of each primer in a tube of 0.5 mL and sent for sequencing (GATC Gmbh, Konstanz, Germany).

### 4.5. Bioinformatic Analysis

Sequence databases were searched with the blast program available https://blast.ncbi.nlm.nih.gov/blast.cgi. The sequence alignments were performed using the Clustalw2 alignment software (http://www.ebi.ac.uk/tools/msa/clustalw2/). The DNA sequences were translated to ORF by expasy translate [42]. The molecular weight and isoelectric point (pI) were predicted by the protParam server [43]. The modeled structure and protein ligand binding site residues of SEC_epi_ were predicted using the I-TASSER server (Iterative Threading ASSEmbly Refinement) in Michigan University [44]. The modeled SEC_epi_ was used for comparison with predetermined structure SEC derived from *S. aureus*.

### 4.6. Cloning of sec_epi_ Gene

The gene fragment corresponding to the mature form of *sec*_epi_ gene was amplified from *S. epidermidis* SE90 DNA using the Phusion High-Fidelity DNA Polymerase (Thermo Scientific, Illkirch-Graffenstaden, France) according to the protocol: 94 °C for 3 min, followed by 30 cycles at 92 °C for 2 min, 50 °C for 1 min, 72 °C for 2 min. The cloning primers including a 5’_ *Bam*HI site, 5’_GAGTCAACCAGACCCTATGCCAGA_3’ and 5’_ *Eco*RI site, 5’_AAGTTTATCCATTCTTTGTTG TAAGGT_ 3’. The amplicon was purified using EZ-10 Spin Column PCR products purification Kit (EUROMEDEX, Souffelweyersheim, France) and DNA fragment digested by *Bam*HI and *Eco*RI was then subcloned into the pGEX-6P-1 glutathione *S*-transferase (GST) fusion expression vector (GE Healthcare, Buc, France). The plasmid containing the intact sequence of the respective region of sec_epi_ was transformed into *Escherichia coli* XL-1 blue, and the insertion of the proper length restriction fragment was verified by plasmid sequencing. The resulting recombinant SEC_epi_ had 5 additional amino acid residues (GPLGS) at the N terminus from pGEX-6P-1. Plasmid DNA midi-preparation was performed, and recombinant plasmid was inserted in *Escherichia coli* BL21 for SEC_epi_ expression and purification.

### 4.7. Expression and Purification of rSEC_epi_

The recombinant clone was used for expression of the gene. Briefly, *Escherichia coli* BL21 harboring recombinant pGEX-6P-1 was grown in 2xTY broth containing 100 µg of ampicillin/mL at 35 °C to an optical density at 600 nm of 0.5. The expression of the GST fusion protein was induced by adding 0.2 M IPTG (isopropyl-D-thiogalactopyranoside, Amersham) to a final concentration of 0.2 mM. After 16 h of cultivation at 27 °C, the cells were harvested and lysed by French Press (French Pressure Cell Press, SLM AMINCO^®^). The clarified lysate was purified by affinity chromatography by using Glutathione Sepharose™ 4B (GE Healthcare, Buc, France). The GST fusion SEC_epi_ was eluted with 10 mM glutathione in 50 mM Tris-HCl (pH 8.0). Mature-form toxins were then released by digestion for overnight at 4 °C with PreScission Protease (GE Healthcare, Buc, France), which cleaves at a single site between the GST tag and the mature form SEC_epi_. The protease and the GST tag was separated by passing the samples through glutathione-sepharose 4B. Purification of rSECepi through a cation-exchange chromatography (40 mM MES, 1 mM DTT, pH 5.7) was further achieved using a NaCl gradient from 0 to 250 mM, to eliminate LPS traces. Purity was verified in Roti^®^-Blue quick-stained sodium dodecyl sulfate polyacrylamide gel electrophoresis (SDS-PAGE) (12% (*w*/*v*)) in agarose gel (0.6% (*w*/*v* of agarose in PBS) against purified *S. aureus* SEC antigens. The protein concentration was assessed by using the Bradford assay (Bio-Rad), Bovine Serum Albumine for calibration and pure mature rSEC_epi_ was stocked at −80 °C until used for biological and biochemical assays. The recombinant SEC from *S. aureus* was obtained from *S. aureus* FRI137 as described in patent PCT/IB2012/050909.

### 4.8. Western Blotting

Purified recombinant protein identity was assess by western blotting as describe [45]. Briefly, the protein concentration was measured using the Bradford reagent (BioRad, Steenvoorde, France) and 50 ng of protein was loaded per well in sodium dodecyl sulfate polyacrylamide gel electrophoresis (SDS-PAGE) (12% polyacrylamide) and transferred to Immobilon-membranes (TransBlot^®^ Turbo™, BioRad, Steenvoorde, France) using the manufacturer’s instructions. After being blocked with 3% bovine serum albumin in phosphate buffered saline (PBS) supplemented with 0.05% (*v*/*v*) Tween 20, membranes were incubated with rabbit affinity-purified anti-*S. aureus* enterotoxin C antibodies (primary antibody) [29,45]. To detect the primary antibody, a goat peroxidase-conjugated anti-rabbit IgG (Sigma-Aldrich, Saint Quentin-Fallavier, France) were used. Proteins were visualized using 4-chloronaphtol (Opti4CN) detection kit according to the manufacturer’s directions (BioRad, Steenvoorde, France).

### 4.9. Determination of Enzyme and Heat Stability of SEC_epi_

SEs stability assays to heat and gastrointestinal environment were performed as reported previously [30]. Bovine serum albumin (BSA, Sigma, St. Louis, MO, USA) and purified *S. aureus* SEC was used respectively as a protein negative control and positive control.

To study the stability of purified protein against heat treatment, 300 µL of the toxin at 3.7 µM in PBS was placed into a heat block maintained at 100 °C. At desired time intervals ranging from 1 to 10 h, tubes were removed from the heat block, immediately put into an ice bath for 5 min to cool down, and then placed at −20 °C.

Purified SEC_epi_, *S. aureus* SEC or BSA, was incubated in the presence of trypsin (Sigma-Aldrich, Saint Quentin-Fallavier, France). Each protein at 3.7 µM was incubated with trypsin (0.04 µM) in 0.01 M Tris-HCl, pH 8.0 in a final volume of 300 mL at 37 °C. After incubation for the desired periods of time ranging from 1 to 10 h, tubes were removed and digestion is stopped by treatment at 95 °C for 5 min. Tubes were immediately stored at −20 °C.

Purified SEC_epi,_ *S. aureus* SEC or BSA was incubated in the presence of pepsin (Sigma-Aldrich, city, France). Each protein at 3.7 µM was incubated with pepsin (0.028 µM) in a final volume of 300 µL of 0.1 M sodium acetate buffer, pH 4.0, at 37 °C. After incubation for the desired periods of time intervals ranging from 1 to 10 h. Tubes were removed, and digestion is stopped by treatment at 95 °C for 5 min. Tubes were immediately stored at −20 °C.

### 4.10. Human Peripheral Blood Mononuclear Cells (hPMBC) Purification and Culture

hPMBC were obtained from samples of heparinized whole blood by centrifugation through a Ficoll-Paque gradient (blood diluted in PBS–Ficoll, 6:4 *v*/*v* [46]. PMBC were resuspended in RPMI 1640 for washing and then recovered by an 800× *g* centrifugation at RT. The cells were resuspended in RPMI medium supplemented with 10% fetal bovine serum, 2 mM de glutaMAX and 0.1% de penicillin/streptomycin. The PMBC were resuspended in 25 mL of complete RPMI, plated in Petri plates, and allowed to incubate 3 h at 37 °C (5% CO_2_). The non-adherent T lymphocyte-enriched cells were collected and washed.

### 4.11. Cell Proliferation Assays

#### 4.11.1. Cell Proliferation with Different Concentrations of SECepi and SECaureus

To assess the mitogenic capacity of toxin, we used the cell proliferation ELISA, BrdU (5-bromo-2-deoxyuridine) colorimetric assay (Roche, Meylan, France) according to the Manufacturer’s recommendations. The purified hPMBC (2 × 10^6^/mL, 0.2 mL) were stimulated with rSEC_epi_ or *S. aureus* SEC at concentration of 3.7 to 3.7 × 10^4^ pM at 37 °C and with or without 100 U of polymyxin B sulfate [Sigma, France/mL, a lipopolysaccharide (LPS) inhibitor] in a 5% CO_2_ incubator. After incubation for 48 h, microplates were supplemented with 10 µM BrdU for 16–24 h. Cell proliferation was estimated by incorporation of BrdU measurement into newly synthesized cell DNA. They were fixed with 200 µL of FixDenat in a microplate for 30 min at 15–25 °C before cells were softly sedimented and resuspended in 100 µL of dividing cells using peroxidase-conjugated anti-BrdU antibodies as recommended by the Manufacturer. After 1 h at room temperature and 3 washes with PBS, the cells are supplemented with TMB. The reaction is stopped after 15 min with H_2_SO_4_ and the absorbances are measured in ELISA reader at 450 nm/690 nm. Data are presented as means of triplicate determinations, as previously described [15].

#### 4.11.2. Cell Proliferation after Toxins SECepi and SECaureus Heating

SECepi et SECaur were diluted at 100 ng/mL in PBS, then heated at 75 °C or 100 °C for 0, 30 or 60 min. Heating effect was evaluated onto the hPBMC-derived T lymphocytes proliferation as described above (2.11.1) in the presence of 37 pM of SECepi or SECaureus according time and heating temperature in triplicate onto independent sources of cells, using the BrdU test. Proliferation is expressed as the absorbance ratio: (DO450/690 assay − DO450/690 control = derived T lymphocytes only).

### 4.12. Screening for Cytokines Production by Activated T Cells

Cytokines interleukin −2 (−4, −6, −8, −10), gamma interferon (IFN-γ), Tumor Necrosis Factor-α (TNF-α) and Granulocyte macrophage colony-stimulating factor (GM-CSF) production in human PBMCs-derived T lymphocytes stimulated by SEC_epi_ and SEC_aureus_ was measured to assess superantigenic activity. Briefly, hPBMCs-derived T lymphocytes were stimulated with 3.7 to 3.7 × 10^3^ pM of purified SEC_epi_. The cells were incubated at 37 °C for 72 h in a humidified 5% CO_2_ atmosphere. Culture supernatants were harvested by centrifugation to cytokine assays. The production of cytokine is determined using Bio-Plex Pro^®^ Human Cytokines 8-plex Assay kit from Bio-Rad Laboratories, Inc. with magnetic beads, and a Bio-Plex 100^®^ System and Bio-Plex Manager^®^ software version 6.0 (Bio-Rad Laboratories, Inc., Hercules, CA, USA) according to manufacturer’s instructions. Briefly, beads were incubated for 20 s on 96 well plates and were washed in Bio-plex wash buffer. Diluted standard and samples were added, and the plates were incubated for 30 min on shaker. After washing, detection antibodies were added and reincubated for 30 min on shaker. The plates were washed and streptavidin-phycoerythrin (SA-PE) added, followed by 10 min of incubation and wash. Assay buffer was finally added and incubated for 10 min, followed by the plates analyzed. Data are presented as the means of standard deviations of triplicate determinations. 

### 4.13. Statistical Analysis

Data are presented as mean ± standard deviation from three independent experiments performed in triplicate. The χ^2^ test was carried out with the Minitab 14 (https://www.minitab.com/academic/) for proportions comparison and the level of significance was set at *p* values < 0.05 for all tests.
